# Two Synchronous Neonatal Tumors: An Extremely Rare Case

**DOI:** 10.1155/2021/6674372

**Published:** 2021-04-19

**Authors:** M. Rodríguez-Zubieta, K. Albarenque, C. Lagues, A. San Roman, M. Varela, D. Russo, G. Podesta, D. Steinberg, C. Schauvinhold, A. Etchegaray, M. T. G. de Dávila

**Affiliations:** ^1^Department of Pathology, Hospital Universitario Austral, Buenos Aires, Argentina; ^2^Department of Oncology, Hospital Universitario Austral, Buenos Aires, Argentina; ^3^Department of Pediatric Surgery, Hospital Universitario Austral, Buenos Aires, Argentina; ^4^Department of Liver Surgery and Transplantation, Hospital Universitario Austral, Buenos Aires, Argentina; ^5^Department of Plastic Surgery, Hospital Universitario Austral, Buenos Aires, Argentina; ^6^Fetal Medicine Unit, Hospital Universitario Austral, Buenos Aires, Argentina

## Abstract

We report a case of a newborn with two synchronous tumors—sialoblastoma and hepatoblastoma—diagnosed at 20 weeks of gestation by magnetic resonance imaging (MRI) and ultrasonography (US). The aim of this study was to describe the management of this case together with a review of the literature. Our patient had a large facial tumor associated with extremely high alpha-fetoprotein levels. Diagnosis of the tumors was made by surgical biopsy, showing typical features in both. Sialoblastoma is a potentially aggressive tumor. In our case, the Ki67 index in the sialoblastoma was between 20 and 30%, indicating a possibly unfavorable behavior. The infant underwent surgery and chemotherapy in different steps. Complete surgical resection with clean margins is considered to be the best treatment option for sialoblastoma. Only four similar cases were previously reported. Timely management by a multidisciplinary team is essential in these difficult cases. In our patient, outcome was good at the time of this report.

## 1. Introduction

Congenital and neonatal salivary gland tumors are exceptionally rare. Sialoblastoma is an uncommon and potentially aggressive tumor. In 1966, Vawter and Tefft [1] reported two cases in neonates and used the term embryoma to describe these tumors. In 1988, Taylor [2] suggested the name sialoblastoma to describe the lesion and this has become the preferred term accepted in the WHO classification [3]. Hepatoblastoma is also a rare tumor in children, comprising less than 1% of pediatric malignancies; however, it is the most common liver malignancy in the neonatal period. Overall, neonatal hepatoblastoma accounts for less than 10% of pediatric hepatoblastoma [4]. We report a boy who was prenatally diagnosed with the unusual association of sialoblastoma and concomitant hepatoblastoma.

## 2. Case Report

**Figure 1:**
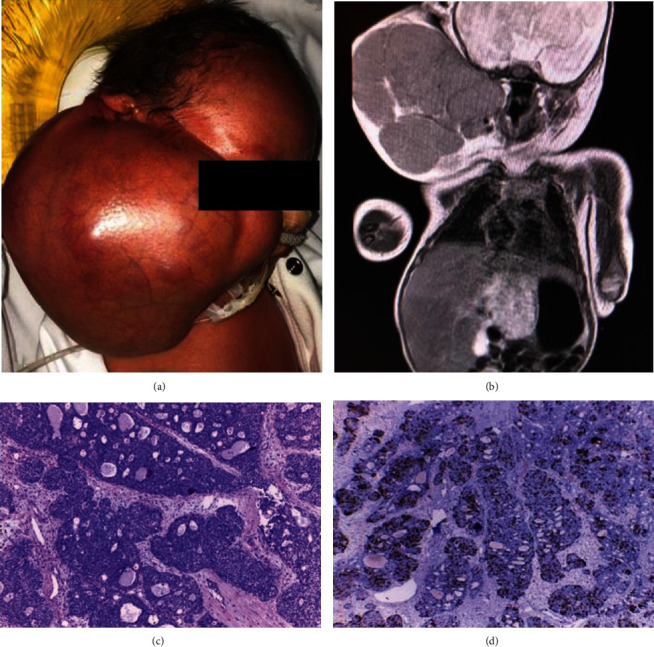
Two synchronous neonatal tumors. (a) Newborn with facial and cervical mass. (b) Magnetic resonance imaging showing cervical multinodular mass and liver tumor. (c) Light microscopy shows sialoblastoma with nests of basaloid (epithelial) cells and a cribriform pattern (H&E, 20x HPF). (d) The proliferative index revealed an increased number of nuclei staining positively with Ki67 (clon Mib-1, immunohistochemistry, 40x HPF).

The patient was a male infant with a prenatal diagnosis of a tumor in the head and neck at 20 weeks of gestation by magnetic resonance imaging (MRI) and ultrasonography (US). Synchronously, a tumor in the caudate segment of the liver was observed. Due to fast growth of the facial lesion during prenatal follow-up and as the infant met the criteria of fetal maturation, delivery was recommended at 34 weeks of gestation. A cesarean section was programmed, and at birth, a large solid mass was seen involving the right hemiface and neck (Figure 1(a)).

The boy was born at 35 weeks of gestation with adequate weight for gestational age. His weight was 3270 g, height 46.5 cm, and head circumference 32.5 cm. Apgar score was 9 and 10 at 0 and 1 minute, respectively.

MRI showed a solid tumor in the right facial area compatible with a soft tissue tumor and liver mass (Figure 1(b)).

Just after birth, the liver lesion was confirmed by computed tomography, showing two satellite lesions and a nodule in the lung suspicious of metastasis. Alpha-fetoprotein (AFP) levels were 365,121 ng/ml (mean normal value 21,135 ng/ml).

On US, a right lateral cervical tumor suggestive of hemangioma was observed. The lesion measured 114 × 74 × 80 mm and spared the airway. Doppler US was normal showing a solid echostructure without cysts. Hemangioma or, less probably, lipoblastoma was considered, among others.

Complete laboratory studies and brain, abdominal, and retroperitoneal US were requested.

MRI of the head showed a solid polylobulated mass with septae occupying the right lateral region of the neck. The lesion enhanced after intravenous gadolinium administration. The morphology and signal enhancement suggested a neural or angiomatous tumor.

On abdominal US, a solid heterogeneous liver mass with a well-defined margin and a peripheral hypoechoic halo was observed anteriorly to the aorta. The mass measured 40 × 28 mm and showed no signal on color Doppler US.

Brain US was normal.

Based on these findings, it was concluded that this patient had two synchronous tumors: a cervical and a liver tumor.

A surgical biopsy of the cervical mass was performed at 5 days of life. The diagnosis of sialoblastoma was confirmed. Histology showed lobulated solid nests and microcystic areas (Figure 1(c)). Perineural invasion was not identified. The proliferation fraction index (Ki67) ranged from 20% to 30% in different areas (Figure 1(d)). Immunoperoxidase staining showed the presence of cytokeratins in the ductal cells as well as vimentin, actin, and S-100 protein in the outermost layer of the ducts. The solid nests of cells were focally reactive to S-100 and vimentin.

**Figure 2:**
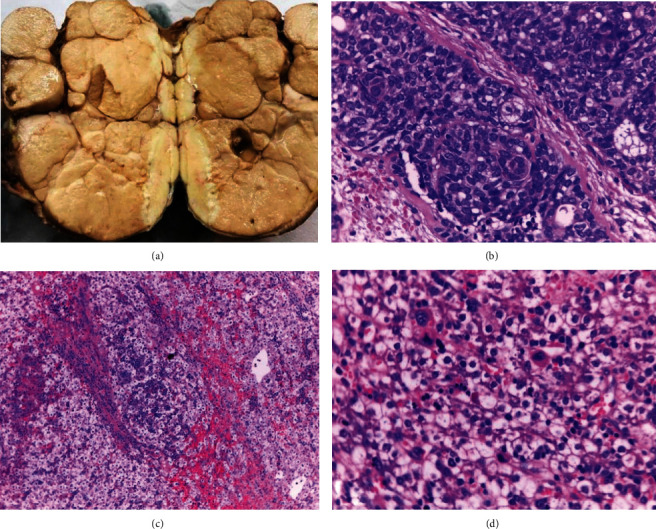
Two synchronous neonatal tumors. (a) Photographs showing the gross appearance of a multinodular mass corresponding to sialoblastoma (11 × 9 × 8.5 cm). (b) Sialoblastoma showing a solid lobular pattern with regular epithelial cells without either mitotic figures or pleomorphism (H&E, 40x HPF). (c) Light microscopy shows fetal and embryonal hepatocytes with a clear and acidophilic cytoplasm (H&E, 20x HPF). (d) Fetal epithelial hepatoblastoma area showing a characteristic pattern (H&E, 40x HPF).

Based on the liver images, the high AFP levels, and the association described in the literature, the liver lesion was considered to be a hepatoblastoma (PRETEXT stage 2M). Chemotherapy was started with VAC (vincristine, actinomycin, and cyclophosphamide) as a less toxic alternative because of the young age of the infant (9 days of life). Due to poor response to the treatment, the liver tumor was surgically removed. No satellite lesions were found on intraoperative US. At 22 days of life, the pediatric liver surgeons resected the liver tumor measuring 2.3 × 1.4 × 2.5 cm. Histology showed epithelial hepatoblastoma of the fetal and embryonal subtype (Figures 2(c) and 2(d)). Immunohistochemistry disclosed positivity for nuclear beta-catenin and cytoplasmatic positivity for hepar-1 and AFP. A mixed epithelial hepatoblastoma with fetal and embryonal compounds was confirmed. After removal of the liver tumor, AFP levels were 78,560 ng/ml. Therefore, chemotherapy based on the SIOPEL 4 strategy was started at 30 days of life with one cycle of cisplatin with marked decrease of the AFP levels (37,895 ng/ml). Nevertheless, the facial tumor did not decrease in size.

At 45 days of life and after recovery from chemotherapy, the sialoblastoma was completely resected. The tumor involved the parotid gland and the facial nerve.

Macroscopy showed a specimen measuring 11 × 9 × 8.5 cm and another specimen of 3 × 3 × 2.5 cm including the parotid gland of 1.8 cm (Figure 2(a)). The tumor had a thin capsule and a tan-yellowish lobulated smooth cut surface. Histology showed solid nests in a cribriform pattern with large luminal spaces and amorphous material inside. The cells had round or ovoid nuclei and were hyperchromatic with mild anisokaryosis and a moderate amount of eosinophilic cytoplasm. In another area, ductal branching and proliferation were seen. No infiltration of the capsule was observed, and the surgical margin was tumor-free (Figure 2(b)). AFP levels were 38.00 ng/ml.

**Figure 3:**
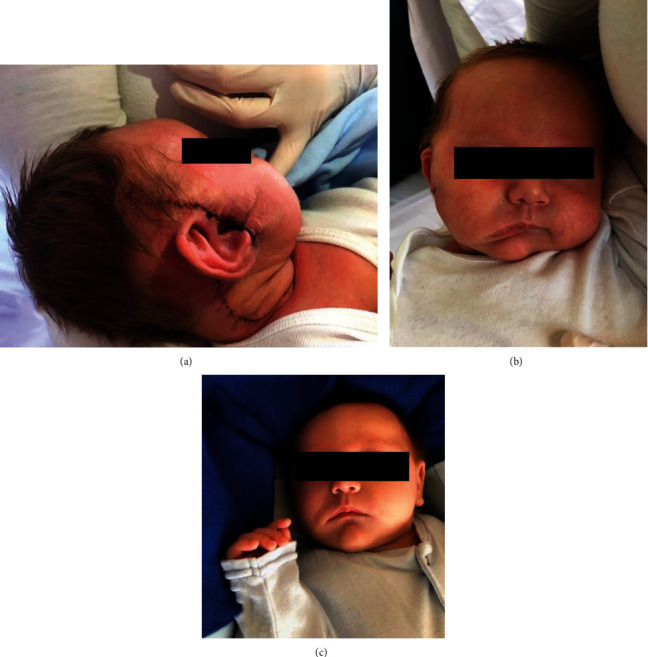
Two synchronous neonatal tumors. Postsurgical photos of the patient: (a) surgical scar; (b, c) mild hemifacial paresis.

After 2 years of follow-up, the general status of the boy was good with only mild hemifacial paresis (Figures 3(a)–3(c)). At this point, he was in complete remission of both tumors on abdominal, thorax, and brain CT scan.

## 3. Discussion

Sialoblastoma is a rare tumor. Currently, in the English literature, 79 pediatric cases of sialoblastoma as a single entity have been published [5]. However, the synchronous association of sialoblastoma and hepatoblastoma is extremely rare, with only four previously reported cases [6–9]. Our case would be the fifth in children younger than 3 years. It has been proposed that because of the common embryonal origin of the parotid gland and the liver (the primitive gut), the abnormality would affect the cells of both organs leading to the cooccurrence of two separate synchronous tumors in the same patient.

Sialoblastoma has also been named embryoma or basaloid cell adenoma. Taylor [2] argued that congenital salivary gland tumors originate from the cells of a primitive blastema and that these lesions are analogous to blastomas that arise from other organs and that are capable of differentiation along more than one cell line. His hypothesis would account for the mixture of cell types observed and reported in the literature as well as the overall within-group similarities. The author suggested to distinguish between *benign* and *malignant* sialoblastoma, depending on histology, cytology, and clinical features.

In the majority of cases, the tumor is located in the parotid gland and less frequently in other salivary glands, with a ratio of approximately 3 : 1. The male to female ratio is 2 : 1. Histologically, the lesion is characterized by nests of basaloid cells with peripheral palisading and adenoid cystic cribriform areas, interspersed within a fibroconnective stroma. The scanty cytoplasm is amphophilic with round-to-oval nuclei. The tumor cells diffusely express S-100, CK19, P63, and *β*-catenin [10, 11].

The mitotic count is variable, ranging from 4 to 12 figures per HPF. Apoptosis and necrosis may be recognized.

Anaplasia, neurovascular invasion, and a Ki67 index of 25-30% were reported to be predictors of a poor prognosis. The histologic parameters of Batsakis and Frankenthaler can be identified as favorable or unfavorable behavior [12]. The incorporation of the Ki67 index as either a favorable or an unfavorable histologic marker may be useful in the prognosis of these tumors [11, 12].

Vascular tumor, lipoblastoma, teratoma, and rhabdomyosarcoma are included in the differential diagnosis. In our case, the initial clinical and imaging diagnosis was a vascular or soft tissue tumor and therefore, MRI and radiologic findings are necessary to suspect the tumor.

Treatment of sialoblastoma involves complete surgical excision with tumor-free margins and chemotherapy previous to surgery to reduce the tumor or when local recurrence (22%) or lung metastasis occurs [3, 5, 13].

Hepatoblastoma is the most common malignant tumor of the liver in childhood; however, incidence is less than 10% in neonates. In our case, the histologic type was epithelial of the mixed embryonal/fetal subtype according to the liver tumor consensus classification [5].

Previously, four similar cases were published in the English literature and one reported in a Korean journal was associated with a chromosome 1q 11p deletion [6] (Table 1).

Hepatoblastoma, pancreatoblastoma, germ-cell tumors, and sialoblastoma are among the embryonal tumors associated with elevated AFP levels. In our case, the AFP levels were increased because the patient had two of these tumors.

The cooccurrence of two synchronous embryonal and AFP-producing tumors is extremely rare, and further knowledge is necessary to define the best approach and treatment options in these patients.

## 4. Conclusion

Our case presented with two embryonal tumors, which makes it of clinical and prognostic interest showing that when sialoblastoma is diagnosed, synchronous cooccurrence of hepatoblastoma should be ruled out. The very high AFP levels warrant evaluation of the abdomen to search for a liver tumor. Proliferation fraction Ki67 should be evaluated as a possible predictive factor for tumor relapse or metastasis. Timely management by a multidisciplinary team is essential in these difficult cases. Outcome of the patient was good at the time of this report.

## Figures and Tables

**Table 1:** Case reports of patients with sialoblastoma and hepatoblastoma.

Case	Author	Year	Journal	Country	Age at Dx	Sex	AFP	Histopathological diagnosis	Other data	Treatment	Follow-up
1	Choi HJ	1999	Korean Obstet Gynecol 42(1):175-8	Korea	N/A	N/A	Elevated	Sialoblastoma & hepatoblastoma	Chr 1q Del	N/A	N/A
2	Siddiqi, SH	2000	Pediatr Radiol 2000,30:349-51	USA	37 weeks	Female	Elevated	Sialoblastoma & hepatoblastoma		Surgery & chemotherapy	Alive 5.5 years postsurgery
3	Stones, DK	2009	Pediatr Blood Cancer 2009;52:883-885	South Africa	34 weeks	Male	Elevated	Sialoblastoma & hepatoblastoma	Postmortem evaluation		Died/sepsis
4	Cheng, YK	2012	Prenat Diagn 2012,9:915-7	China	28 weeks	Male	Elevated	Sialoblastoma & hepatoblastoma		Surgery & chemotherapy	Alive 8 months postsurgery
5	G de Dávila	2018		Argentina	20 weeks	Male	Elevated	Sialoblastoma & hepatoblastoma		Surgery & chemotherapy	Alive 31 months postsurgery

Abbreviations: AFP: alpha-fetoprotein; N/A: not available.
